# Implementation of PET/CT in radiation oncology—a patterns-of-care analysis of the German Society of Nuclear Medicine and the German Society of Radiation Oncology

**DOI:** 10.1007/s00066-024-02260-4

**Published:** 2024-08-09

**Authors:** Simone Wegen, Ursula Nestle, Constantinos Zamboglou, Simon K. B. Spohn, Nils Henrik Nicolay, Lena M. Unterrainer, Stefan A. Koerber, Christian La Fougère, Emmanouil Fokas, Carsten Kobe, Chukwuka Eze, Anca-Ligia Grosu, Wolfgang P. Fendler, Adrien Holzgreve, Rudolf Werner, Nina-Sophie Schmidt-Hegemann

**Affiliations:** 1 Center for Integrated Oncology Aachen Bonn Cologne Duesseldorf (CIO ABCD), Faculty of Medicine and University Hospital Cologne, University of Cologne, Cologne, Germany; 2 Department of Radiation Oncology, Kliniken Maria Hilf, Mönchengladbach, Germany; 3 Department of Radiation Oncology, University Hospital Freiburg, Freiburg, Germany; 4 Department of Radiation Oncology, University Hospital Leipzig, Leipzig, Germany; 5 Department of Nuclear Medicine, LMU University Hospital, LMU Munich, Munich, Germany; 6 Department of Radiation Oncology, Heidelberg University Hospital, 69120 Heidelberg, Germany; 7 Department of Radiation Oncology, Barmherzige Brüder Hospital Regensburg, Regensburg, Germany; 8 Nuclear Medicine and Clinical Molecular Imaging, Department of Radiology, Eberhard Karls University of Tübingen, Tübingen, Germany; 9 Department of Nuclear Medicine, Faculty of Medicine and University Hospital Cologne, University of Cologne, Cologne, Germany; 10 Department of Radiation Oncology, LMU University Hospital, LMU Munich, Munich, Germany; 11 German Cancer Consortium, German Cancer Research Center, Freiburg, Germany; 12 Department of Nuclear Medicine, University Hospital Essen, Essen, Germany; 13 Divison of Nuclear Medicine, University Hospital, Goethe University Frankfurt, Frankfurt, Germany; 14 German Cancer Consortium, German Cancer Research Center, Freiburg, Germany; 15 PET committee of the German Society of Nuclear Medicine, (DGN), Germany; 16 Department of Radiation Oncology, Cyberknife and Radiotherapy, University Hospital Cologne, Cologne, Germany

**Keywords:** PET/CT, Planning PET, Survey, Germany, Reimbursement

## Abstract

**Background:**

The use of positron-emission tomography (PET)/computed tomography (CT) in radiation therapy (RT) has increased. Radiation oncologists (RadOncs) have access to PET/CT with a variety of tracers for different tumor entities and use it for target volume definition. The German Society of Nuclear Medicine (DGN) and the German Society of Radiation Oncology (DEGRO) aimed to identify current patterns of care in order to improve interdisciplinary collaboration.

**Methods:**

We created an online survey on participating RadOncs’ use of PET tracers for different tumor entities and how they affect RT indication, dose prescription, and target volume definition. Further topics were reimbursement of PET/CT and organizational information (fixed timeslots and use of PET with an immobilization device [planning/RT-PET]). The survey contained 31 questions in German language (yes/no questions, multiple choice [MC] questions, multiple select [MS] questions, and free-text entry options). The survey was distributed twice via the DEGRO member mailing list.

**Results:**

During the survey period (May 22–August 7, 2023) a total of 156 RadOncs (13% of respondents) answered the survey. Among these, 59% reported access to diagnostic PET/CT within their organization/clinic and 24% have fixed timeslots for their patients. 37% of survey participants can perform RT-PET and 29% have the option of providing a dedicated RT technician for planning PET. Besides [^18^F]-fluorodeoxyglucose (FDG; mainly used in lung cancer: 95%), diagnostic prostate-specific membrane antigen (PSMA)-PET/CT for RT of prostate cancer is routinely used by 44% of participants (by 64% in salvage RT). Use of amino acid PET in brain tumors and somatostatin receptor PET in meningioma is low (19 and 25%, respectively). Scans are reimbursed through private (75%) or compulsory (55%) health insurance or as part of indications approved by the German Joint Federal Committee (*Gemeinsamer Bundesausschuss*; 59%). 98% of RadOncs agree that PET impacts target volume definition and 62% think that it impacts RT dose prescription.

**Discussion:**

This is the first nationwide survey on the role of PET/CT for RT planning among RadOncs in Germany. We find high acceptance of PET results for treatment decisions and target volume definition. Planning PET comes with logistic challenges for different healthcare settings (e.g., private practices vs. university hospitals). The decision to request PET/CT is often based on the possibility of reimbursement.

**Conclusion:**

PET/CT has become an important tool for RadOncs, with several indications. However, access is still limited at several sites, especially for dedicated RT-PET. This study aims to improve interdisciplinary cooperation and adequate implementation of current guidelines for the treatment of various tumor entities.

## Introduction

The significance of positron-emission tomography/computed tomography (PET/CT) in radiotherapy (RT) treatment planning is increasing, and PET/CT, since its introduction, has markedly improved the clinical management of cancer patients [1, 2]. In specific tumor entities, [18F]-fluorodeoxyglucose (FDG) PET/CT has become firmly established as a pivotal tool for tumor detection and target volume delineation [3]. In patients with prostate cancer, prostate-specific membrane antigen (PSMA)-PET/CT-guided RT proves invaluable for initial tumor staging, local recurrence, and detection of oligometastatic disease (≤ 5 lesions) or metastatic progression, thus playing a crucial role in treatment planning [4–6]. Advantages of PET-guided RT encompass a potentially higher total RT dose (e.g., ablative treatment of metastasis) and dose reduction in surrounding normal tissue (sparing organs at risk, OAR) [7]. The 2008 IAEA Technical Documentation series (IAEA TECDOC-1603) delves into the role of PET/CT in treatment planning for cancer patients, presenting early evidence to support its use in head and neck cancers, esophageal cancer, cervical and colorectal cancer, and malignant melanoma [8].

According to the German Joint Federal Committee (*Gemeinsamer Bundesausschuss* [G-BA]), in the version dated January 17, 2006, last amended October 20, 2022, and entering into force on January 14, 2023, PET may be performed for the following indications at the expense of statutory health insurance (summary for radiotherapy-relevant indications) [9]:

[^18^F]FDG PET/CT:

- Determination of tumor stage in primary non-small cell lung cancer (NSCLC) including detection of distant metastases and detection of tumor recurrence (in cases of reasonable suspicion).
- Assessment of solitary pulmonary nodules in patients with an increased surgical risk and when a diagnosis cannot be made using invasive methods.
- Determination of tumor stage in small cell lung cancer (SCLC) with a curative approach and detection of tumor recurrence (in cases of reasonable suspicion).
- Staging examinations for Hodgkin’s lymphoma in adults with initial disease and relapsed disease; not for follow-up of patients without justified suspicion of recurrence.
- Patients with locally advanced head and neck cancer or cancer of unknown primary (CUP) prior to the decision on the performance of neck dissection.
- Patients with reasonable suspicion of persistent disease or recurrence of laryngeal cancer after curative treatment to decide on laryngoscopic biopsy.
- Malignant lymphoma in children and adolescents.
- Initial staging of aggressive non-Hodgkin’s lymphoma.

[^68^Ga]/[^18^F]-labeled PSMA PET/CT:

- Primary staging in high-risk prostate cancer and patients with suspicion of recurrent disease (rise in PSA).

Somatostatin-receptor PET/CT (e.g., 1,4,7,10-tetraazacyclododecane-tetraacetic acid–octreotate ([^68^Ga]Ga-DOTATATE):

- In meningioma and in gastrointestinal neuroendocrine tumors (GI-NET; here only as part of the outpatient specialist care network [*ambulante spezialfachärztliche Versorgung*, ASV]); somatostatin-receptor PET/CT in meningioma is not included in the G‑BA approval.

Amino acid PET/CT (e.g., [^18^F]-fluoroethyltyrosine (FET):

- For detection and differentiation of brain tumors; amino acid PET/CT is not included in the G‑BA approval and the reimbursement of costs is at the discretion of the responsible health insurance company.

The accessibility of PET/CT services in Germany encompasses facilities such as university hospitals, clinics, and private practices offering PET/CT scattered across the country. This ensures timely and accurate diagnoses, contributing to improved outcomes in the management of cancer patients. Germany’s healthcare system ensures that PET/CT services are available to a broad spectrum of the population for the abovementioned indications.

To the best of our knowledge, there have been no data collected on the use of PET/CT among radiation oncologists (RadOncs) in Germany. The German Society of Nuclear Medicine (DGN) and the German Society of Radiation Oncology (DEGRO) aim to identify current patterns of care in order to improve interdisciplinary collaboration. We here present the results of a nationwide online survey.

## Materials and methods

The survey entitled “PET/CT in radiotherapy—a patterns-of-care analysis by the Nuclear Medicine and Radiotherapy Working Group of DEGRO and the DGN” was developed in collaboration with German RadOncs and nuclear medicine physicians. It was created between January and April 2023 using the SurveyMonkey platform (a global survey platform, based in San Mateo, California, USA) (https://www.surveymonkey.com) and contained 31 questions in German language (yes/no questions, multiple choice [MC] questions, multiple select [MS] questions, free-text entry options). Topics were the surveyed person’s background (professional status, working place), clinical indications RadOncs see for PET/CT in different tumor entities, how PET/CT is reimbursed, and whether PET/CT affects target volume delineation and dose finding. Participation was anonymous and voluntary. The survey could be interrupted or cancelled at any time. A pretest was carried out in a group of four colleagues working in radiation oncology and nuclear medicine to check the comprehensibility, accessibility, and technical compatibility of the survey and to make appropriate corrections.

The link to the online survey and a reminder were distributed via the DEGRO members’ mailing list (1232 DEGRO members). The first email round was on May 22, 2023, and a reminder was sent on June 14, 2023. We presented a QR code (scannable by mobile devices) with a call for participation during the working group (*AG Nuklearmedizin/Strahlentherapie*) meeting at the 29th Annual Congress of the DEGRO on June 22, 2023. Here, interim results of the survey were presented. The survey period was May 22–August 7, 2023. All questions were provided in the German language and placed on SurveyMonkey (https://www.surveymonkey.de/r/AG_NUK_STH). All responses were checked for completeness and collected in an Excel (Microsoft) table. Responses to open-ended questions were collected separately. Due to the heterogeneity of the data collected, we decided to perform a descriptive analysis only (see the “Limitations” section). We present results along the lines of the below-mentioned main categories.

## Results

### Characteristics of surveyed participants

Between May 7 and August 10, 2023, 156 participants from all 16 federal states of Germany answered the survey. Table 1 shows the participants’ characteristics: 36% indicated working in an outpatient radiation oncology facility, 26% in a medical care center (*Medizinisches Versorgungszentrum* [MVZ]; MVZs can be tied to a hospital [academic/nonacademic] or detached from the hospital care system), and 26% in a university hospital. Radiation oncology specialist (*Facharzt*/*Fachärztin*) was the most common function (40%), 22% work as senior physicians (*Oberarzt*/*Oberärztin*), and 20% work as head physicians (*Chefarzt*/*Chefärztin*); 12% of those surveyed were in training (*Assistenzarzt*/*Assistenzärztin*; see Table 1).

**Table 1:** Characteristics of participants

	Overall (*N* = 156)
*State (Bundesland)*	
North Rhine-Westphalia	42 (26.9%)
Baden-Württemberg	20 (12.8%)
Bavaria	16 (10.3%)
Hesse	11 (7.1%)
Saxony	10 (6.4%)
Berlin	7 (4.5%)
Lower Saxony	7 (4.5%)
Rhineland-Palatinate	7 (4.5%)
Hamburg	4 (2.6%)
Schleswig-Holstein	4 (2.6%)
Mecklenburg-Western Pomerania	3 (1.9%)
Thuringia	3 (1.9%)
Bremen	2 (1.3%)
Brandenburg	1 (0.6%)
Saarland	1 (0.6%)
Saxony-Anhalt	1 (0.6%)
Missing	17 (10.9%)
*Professional role*	
Specialist (*Fachärztin*/*Facharzt*)	60 (38.5%)
Senior physician (*Oberärztin*/*Oberarzt*)	33 (21.2%)
Head physician (*Chefärztin*/*Chefarzt*)	30 (19.2%)
Resident/in training (*Assistenzärztin*/*Assistenzarzt*)	18 (11.5%)
Other^a^	7 (4.5%)
Missing	8 (5.1%)
*Workplace*	
Radiooncology practice/ambulatory health care center (*Strahlentherapeutische Praxis*/MVZ)	55 (35.3%)
Clinic (± ambulatory health care center; *Klinik, ggf. mit MVZ*)	51 (32.7%)
University hospital (*Universitätsklinik*)	40 (25.6%)
Other^b^	2 (1.3%)
Missing	8 (5.1%)

^a^Other: supervisory board (*Aufsichtsrat*), specialist (*Fachärztin*/*Facharzt für Strahlentherapie*), proprietor (*Inhaber:in*), technician (MTR), management of medical care center (*MVZ Leitung, Geschäftsführung*), physicist (*Physiker:in*), site manager (*Standortleiter:in*)

^b^Other: Industry (Industrie), medical service of the health insurance funds (*Medizinischer Dienst der Krankenkassen*, MDK)

### Purpose of PET/CT

**Fig. 1:**
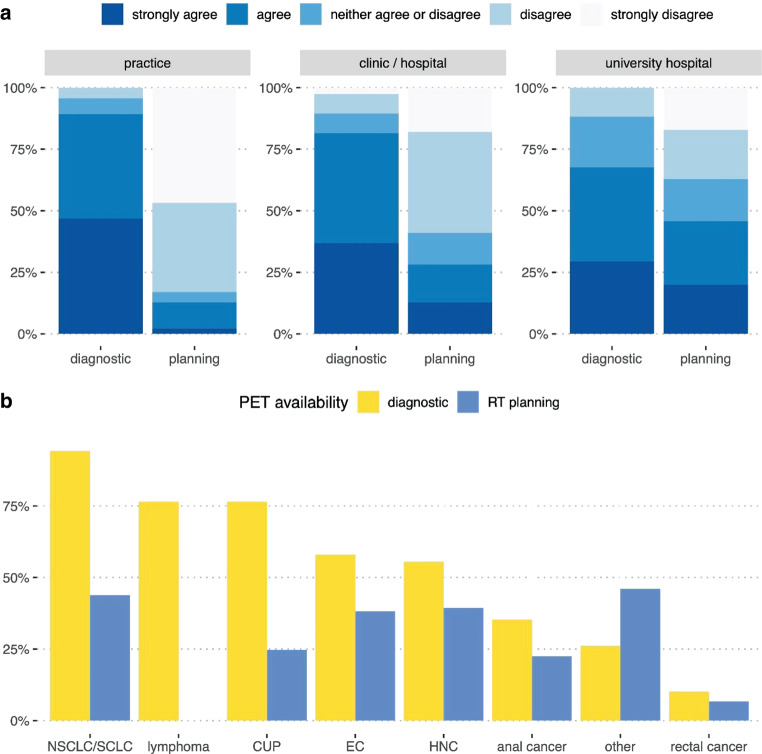
Use of FDG PET. **a** Statement 1: “In the practice/clinic/hospital I work at, I can request FDG PET/CT as a *diagnostic* tool prior to radiotherapy.” Statement 2: “In the practice/clinic/hospital I work at, I can request FDG PET/CT as *planning* PET/CT (in RT position, with presence of a medical radiation technologist) prior to radiotherapy.” Likert scale, divided by working place; **b** FDG PET/CT requested by RadOncs in the diagnostic setting (*yellow*) and for RT planning (*blue*) according to different tumor entities

The majority of PET/CTs requested by RadOncs were reported to be diagnostic scans (question [Q]5). Technically, 55% indicated fusing PET/CT scans with the planning CT in their contouring software. On the Likert scale, the rates for “strongly agree” and “agree” for RadOncs performing *diagnostic* PET/CT were highest in participants from practices with an outpatient medical care center (MVZs; 47 and 43%, respectively) and lowest among members from university hospitals (29 and 38%, respectively; Fig. 1a). Rates for *planning* PET/CT (performed in the planning position with a medical radiation technologist [*Medizinischer Technologe für Radiologie*, MTR] present, using positioning devices, thermoplastic masks, etc.) were highest in university hospitals (“strongly agree” 20%, “agree” 26%) and lowest in outpatient radiation oncology facilities (2 and 11%, respectively; Q6).

### [18F]FDG PET/CT

According to the multiple select question (Q8), the most frequently requested indications for diagnostic [^18^F]FDG PET/CT were lung cancer (94%), CUP (76%), lymphoma (76%), esophageal cancer (58%), and head and neck cancer (HNC; 55%; Fig. 1). The overall rates for PET/CT in the treatment position (Q9) were lower: in lung cancer PET/CT was indicated by 44% of RadOncs, in HNC by 39%, and in esophageal cancer by 38%. In the free-text entry option, the use of [^18^F]FDG PET/CT was mentioned particularly for gynecological malignancies, i.e., cervical cancer (mentioned five times).

### [^68^Ga]/[^18^F]PSMA PET/CT

**Fig. 2:**
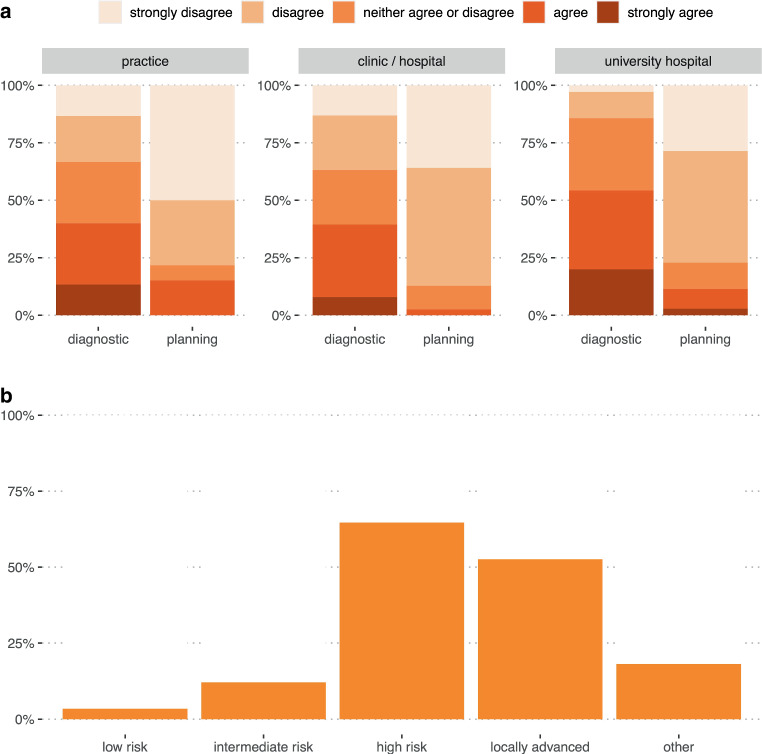
Use of PSMA PET. **a** Statement 1: “In the practice/clinic/hospital I work at, I can request PSMA PET/CT as a *diagnostic* tool prior to radiotherapy.” Statement 2: “In the practice/clinic/hospital I work at, I can request PSMA PET/CT as a *planning* PET/CT (in RT positioning, with the presence of a medical radiation technologist) prior to radiotherapy.” Likert scale, divided by working place; **b** PSMA PET/CT (diagnostic and planning) requested according to prostate cancer tumor stage

In primary prostate cancer, 14% of participating RadOncs “fully agreed” and 31% “agreed” on the routine use of *diagnostic* PSMA PET/CT, while 19% “disagreed” and 10% “strongly disagreed” with using diagnostic PSMA PET/CT ahead of radiotherapy (Q10; Fig. 2a). The heterogeneity of answers is underpinned by 27% neither agreeing nor disagreeing to the regular use of diagnostic PSMA PET/CT in primary prostate cancer. When asked for *planning* PSMA PET/CT, the overall rates for requesting PET/CT were lower (Q11; Fig. 2a). In the subgroup analysis, participants from university hospitals most frequently request *diagnostic* PSMA PET/CTs (“strongly agree” 20%, “agree” 34%). Diagnostic PSMA PET was less frequently requested by RadOncs working in nonuniversity clinics (“strongly agree” 8% and “agree” 32%) and practices (“strongly agree” 13% and “agree” 27%). When using PSMA PET/CT (both diagnostic and planning), 3% of participants request it in “low-risk,” 12% in “intermediate-risk,” 65% in “high-risk,” and 53% in locally advanced prostate cancer. Here, in the free-text entry option, 8 participants mentioned oligometastatic disease as a relevant indication for PSMA PET/CT (Q12, see Fig. 2b).

In biochemical recurrence, before planned salvage radiotherapy, 19% “strongly agree” and 45% “agree” on the use of PSMA PET (*diagnostic*), but this was not true for RT *planning* PET/CT (34% “disagree” and 40% “strongly disagree”; Q13).

### Amino acid and somatostatin PET/CT

The use of amino acid PET/CT (e.g. [^18^F]FET-PET) as a *diagnostic* tool in neurooncological tumors is low (> 60% disagree or strongly disagree with using it, Q14), and even fewer survey participants perform it in RT planning position (87% rate of disagreement and strong disagreement, Q15). In meningioma, we found comparably low rates for the routine use of somatostatin (e.g. [^68^Ga]Ga-DOTATATE PET/CT; Q17), with 19.3% of participants agreeing to use it in the diagnostic setting vs. 54% disagreeing and strongly disagreeing. Again, the rate of RadOncs using somatostatin *planning* PET/CT was lower (78% disagree and strongly disagreeing with its use; Q18).

### Other tracers

The section “In addition, the following tracers are available to us as diagnostic examinations prior to radiation planning (please specify)” revealed a fairly wide distribution of fibroblast activation protein inhibitor (FAPI)-directed PET/CT (mentioned 11 times), C‑X‑C motif chemokine receptor type 4 (CXCR4)-directed [68Ga]Ga-PentixaFor (twice), 18F-sodium fluoride (NaF; once), [^18^F]-fluoromisonidazole ([^18^F]FMISO; once), [^11^C]-choline (once), and [18F]-6-fluoro-L‑3,4‑dihydroxyphenylalanine ([^18^F]DOPA) PET/CT; once; Q20).

### Access to PET/CT

**Fig. 3:**
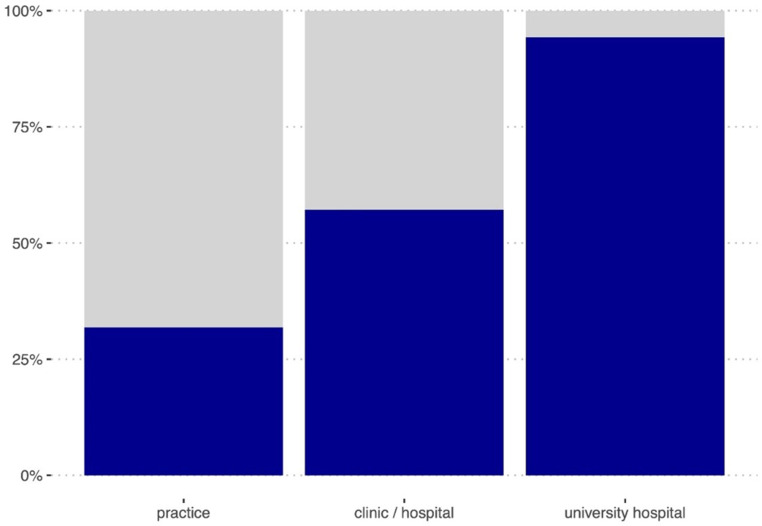
Access to PET/CT stratified by working place. Q22: “I have access to PET/CT within my department/practice network/clinic/university hospital.” Participants answering “yes”: *blue* part of the column; “no”: no data, *grey*; see also Table 2

59% of survey respondents indicated having access to PET/CT in their center or through an association (Q22; Table 2 and Fig. 3). The majority of surveyed RadOncs (76%) do not have fixed timeslots (days/weeks/hours) for their patients in their corresponding nuclear medicine department (Q23) and indicate not running PET/CTs in the planning position (“no” 62%; Q24). 29% of those surveyed indicated having a dedicated MTR or RadOnc physically present in the nuclear medicine department for planning PET. In further analysis, access to PET/CT was highest among participants from university hospitals (83%) and lowest in private practices (26%; Fig. 3).

**Table 2:** Availability of PET

	Radiooncology practice/medical care center (*N* = 55)	Clinic, possibly with outpatient medical care center (*N* = 51)	University Hospital (*N* = 40)	Overall (*N* = 146)
*Access to PET/CT*				
I have access to PET/CT at my disposal in the hospital/clinic/MVZ/practice network	14 (31.8%)	20 (57.1%)	33 (94.3%)	67 (58.8%)
I have fixed days/timeslots with our colleagues in nuclear medicine for carrying out PET/CTs	4 (9.1%)	7 (20%)	16 (45.7%)	27 (23.7%)
We can carry out PET/CTs in the radiation planning position	6 (13.6%)	17 (48.6%)	20 (57.1%)	43 (37.7%)
A radiation therapy technician (MTR) and/or RadOnc is present when PET/CT is performed in the radiation position	1 (2.3%)	16 (45.7%)	16 (45.7%)	33/113 (28.9%)
*Reimbursement*				
Reimbursement is possible via the patient’s compulsory health insurance (*gesetzliche Krankenversicherung*, GKV)	24/42 (57.1%)	14/35 (40.0%)	22/33 (66.7%)	60/110 (54.5%)
Reimbursement is possible via the patient’s private health insurance (*private Krankenversicherung*, PKV)	31/42 (73.8%)	29/35 (82.9%)	22/33 (66.7%)	82/110 (74.5%)
Reimbursement is possible via internal cost allocation with the nuclear medicine department (*interne Leistungsverrechnung*)	3/42 (7.1%)	7/35 (20.0%)	14/33 (42.4%)	24/110 (21.8%)
Reimbursement of costs within the scope of the indications approved by the Federal Joint Committee (G-BA)	21/42 (50.0%)	26/35 (74.3%)	18/33 (54.5%)	65/110 (59.1%)

### Contouring

For contouring purposes, a large majority (92%) of RadOncs routinely import the PET/CT datasets into their contouring software (Q27). Most RadOncs perceive PET/CT as having an influence on target volume delineation (“yes”: 98% of participants; Q29) and to affect total irradiation dose (“yes”: 62% of participants; Q30). When specifically asked how PET information is derived for contouring other than by directly loading the scan into the planning software (see above; Q27), 24% of those surveyed described opening the PET/CT scan and the contouring software on two different screens, 37% discuss target volumes with their nuclear medicine physicist, and 9% make use of autocontouring (within or out of the treatment planning system; Q31).

### Reimbursement

In the corresponding question (Q21; multiple select), 55% of participants indicated getting PET scans reimbursed via the patient’s compulsory health insurance (*gesetzliche Krankenversicherung* [GKV]), e.g., as a university outpatient flat rate (*Hochschulambulanzpauschale*), 75% receive compensation through a patient’s private health insurance (*private Krankenversicherung* [PKV]), and 22% have the option of internal cost allocation (*interne Leistungsverrechnung*) with the nuclear medicine department of their association/hospital. 59% of participants indicated having the option to receive compensation for PET scans according to the list of indications approved by the Joint Federal Committee (G-BA). In the free-text option, 13 participants mentioned reimbursement through outpatient specialist care (*Ambulante Spezialfachärztliche Versorgung* [ASV]) according to the German Social Code § 116b SGB V. Differences in reimbursement according to working place (university hospital vs. clinic/hospital vs. outpatient care/practice) are illustrated in Table 2.

## Discussion

This manuscript presents a comprehensive analysis of the use of PET/CT in RT planning among RadOncs in Germany. The study outlines the current landscape of PET/CT utilization according to clinical indication and addresses issues related to reimbursement, access, and the impact on treatment planning. The survey was sent to all members of the German Association for Radiation Oncology (DEGRO; 1232 members in the mailing list), with 156 participants (13%) answering the survey. Unfortunately, three of Germany’s less densely populated states are only represented by one participant each (Brandenburg, Saarland, and Saxony-Anhalt). Due to the heterogeneity of the collected data, the authors decided to mainly perform a descriptive analysis. Limitations stem from the lack of data on PET/CT use among RadOncs before this survey (no longitudinal comparison over the years) and the fact that there are hardly any comparable surveys from other countries.

During the past decade, impactful trials have been performed on the use of different PET tracers for RT planning, such as the GLIAA trial (amino acid PET in patients with recurrent glioblastoma; NCT01252459; Oehlke et al. [10]). In a pilot prospective trial (GLIAA pilot), the authors found [^18^F]FET PET together with MRI to be most suitable for contouring recurrent glioblastoma tumor tissue [11]. The prospective randomized multicentric PET-PLAN trial (NSCLC contouring based on FDG PET/CT; NCT00697333; Nestle et al. [3]) demonstrated improved local control after PET-based contouring (target volume reduction) without increased treatment-related toxicity. The German Hodgkin Study Group (GHSG) trials HD16, HD17, and HD18 (PET-guided indication for RT, PET-guided contouring; NCT00736320, NCT01356680, NCT00515554 [12–15]) investigated involved-site RT and demonstrated that PET-guided treatment decision making even impacts patients’ outcome and quality of life (QoL): further analyses of the HD18 trial reported faster recovery from fatigue and faster return to work in Hodgkin lymphoma patients when treatment decisions (RT, chemotherapy, immunotherapy) are made based on PET [16]. In meningioma, the use of [11C]-methionine (MET-PET) in target volume delineation was superior to conventional imaging (CT, MRI; Grosu et al. [17]).

For PSMA PET there is a 2021 German survey evaluating the acceptance and use of PET/CT in clinical routine for RT as well as its impact on target volume definition and dose prescription (Vogel et al. [18]). The group found an overall accessibility to PSMA PET in 78% of participants, which is higher than the numbers in our survey (“direct access within my practice/clinic/university hospital” [for all tracers, not specifically PSMA]: 59% of surveyed). An explanation for this discrepancy could be that the 2021 trial participants were not explicitly asked whether their access to PET is within their own practice/clinic/institution (which was a criterion in the corresponding question in our survey). In the literature and in clinical practice, the value of PSMA PET/CT in primary high-risk prostate cancer and oligometastatic and recurrent disease (see also the PSMA SRT trial) is high [19–24].

There are ongoing trials on PET-guided dose escalation in glioblastoma (PRIDE—PRotective VEGF Inhibition for Isotoxic Dose Escalation in Glioblastoma; NCT05871021; ARO 2022-12; PI: Prof. M. Niyazi), in NSCLC (PACCELIO—FDG-PET based small-volume accelerated immunochemoradiotherapy in locally advanced NSCLC; NCT06102057; ARO 2023-06; PI: Prof. U. Nestle), in HNC (INDIRA-MISO trial, radiation dose prescription in HNC based on F‑MISO-PET hypoxia imaging; NCT03865277; PI: Prof. M. Krause), and in prostate cancer (HypoFocal SBRT: PSMA-PET/MRI-Based Focal Dose Escalation in Patients with Primary Prostate Cancer Treated with Stereotactic Body Radiation Therapy; PI: Prof. A.-L. Grosu) [25]. In recent years, any new developments in injectable tracers have soon found their way into radiooncological use (PSMA, FAPI), strengthening the close link between radiotherapy and nuclear medicine [26–31].

In our analysis, the proportion of PET/CTs conducted in the planning position is currently low, despite existing data and recommendations supporting enhanced alignment and reduced radiation exposure through the combination of PET and planning CT [32, 33]. One factor contributing to the limited use of planning PET/CT is likely the requirement for the presence of an MTR in the nuclear medicine department. In our survey, 38% of participants indicated performing planning PET/CT, but only 29% stated actually having an MRT physically present in the nuclear medicine department, also to help with positioning. Thus, one can presume that a significant number of planning PET/CTs are performed without a dedicated MTR, probably reducing the quality of reproducibility and thus the quality of the subsequent radiotherapy. It should be acknowledged that during their education, MTRs are equally trained in radiology, nuclear medicine, and radiotherapy. Thus, there might be expertise of radiotherapy MTRs in PET departments, particularly in the MVZ setting of clinics/practices with various radiation disciplines. However, technicians from specialized units (e.g., nuclear medicine) often lack the confidence to perform tasks such as patient positioning and immobilization, especially if they have been away from the joint training program for a significant length of time. We see potential for improvement through accessible refresher courses at annual congresses and conferences, which would enhance skills and confidence.

In pelvic/urogenital tumors like prostate or cervical cancer [34], a disadvantage in employing planning PET/CT is due to an overshadowing PET tracer accumulation in the full bladder (patients are routinely advised to have a full bladder in the planning CT), compromising the visibility and differentiation of the primary tumor region (in cervical, prostate, rectal, or anal cancer patients). Further logistical challenges arise when patients requiring thermoplastic masks (HNC, brain tumors) need to have these created in a separate appointment before the PET scan (additional work for staff and patients). Interestingly, in our analysis, the overall acceptance rate of diagnostic FDG PET/CT is highest in practices, outpatient clinics, and medical care centers (MVZs) and lower in university hospitals (“strongly agree” and “agree” rate in practices: 47 and 43% vs. 29 and 38% in university hospitals, respectively). Rates for *planning* PET/CTs are highest in university hospitals; however, we suspect that in our survey, the terms “planning PET-CT” and “diagnostic PET-CT” were misleading, leading to the abovementioned heterogeneity in answers. The term “planning PET” refers to scans conducted in planning position. The corresponding questions in the survey had a note saying “PET/CT in planning position, with immobilization devices and a technician/RadOnc physically present during the scan.” However, we suppose that some participants could have interpreted “planning PET-CT” as referring to examinations done in preparation for radiation therapy but not explicitly performed in planning positions. This may have contributed to the unexpected variations in response patterns observed between private practices and university hospitals.

There is a list of indications approved by the Joint Federal Committee (G-BA) for financial compensation (reimbursement) of PET/CTs within the scope of compulsory health insurance (GKV), private health insurance (PKV), and via internal cost allocation (*interne Leistungsverrechnung*) between different departments in one hospital association. The catalog of indications and recommendations is continuously expanded, with PET constantly reassessed by the G‑BA. Detached from this, the outpatient specialist care (*Ambulante Spezialfachärztliche Versorgung* [ASV]) according to Social Code § 116b SGB V offers another option for financial compensation of PET/CT in patients with gynecological (except breast), urological, thoracic, head and neck, skin, brain/neurooncological, and gastrointestinal malignancies as well as sarcoma for practices and hospitals participating in the ASV network.

Possibly, a survey similar to this one directed at nuclear medicine (NucMed) physicians could provide further insights and answer questions regarding referral of patients from RadOnc departments, the availability of tracers within the PET centers or the complexity of reimbursement, which remains a significant barrier to PET availability in Germany. The results collected in this survey may be biased by the exclusive RadOnc perspective. The RadOnc/NucMed working group (*AG Nuklearmedizin/Strahlentherapie der DGN und DEGRO*) is considering setting up a second survey among NucMeds to answer these questions.

In our survey, NSCLC, lymphoma, and CUP were the most frequent tumor entities for requesting PET/CT in a radiooncology context and among these entities, reimbursement is considered certain. National and international guidelines show good evidence for PET/CT in head and neck tumors and esophageal cancer, for instance, but in Germany, these indications can only be reimbursed in the context of ASV care [35, 36]. To counteract the tendency of establishing the indication for PET/CT depending on the probability of financial reimbursement and not primarily on the basis of current guidelines or treatment recommendations, the German Society of Nuclear Medicine (DGN) and the German Society of Radiation Oncology (DEGRO) need to improve interdisciplinary collaboration and work on transparency in terms of reimbursement.

## Conclusion

This nationwide survey reveals high acceptance and increasing integration of PET/CT in RT planning, especially for [^18^F]FDG and PSMA-PET/CT. Nevertheless, the rates of dedicated planning PET/CTs and fixed timeslots for RadOncs in nuclear medicine departments are still low. The decision to request PET/CT is often based on the possibility of reimbursement. The aim of the DGN-DEGRO working group is to further help to adequately implement current guidelines on PET use for the treatment of various tumor entities and to get these indications reimbursed.
